# Co-Extraction and Co-Purification Coupled with HPLC-DAD for Simultaneous Detection of Acrylamide and 5-hydroxymethyl-2-furfural in Thermally Processed Foods

**DOI:** 10.3390/molecules24203734

**Published:** 2019-10-16

**Authors:** Jiaqi Shi, Zeping Shao, Honglei Li, Yan Zhang, Shuo Wang

**Affiliations:** 1 Tianjin Key Laboratory of Food Science and Health, School of Medicine, Nankai University, Tianjin 300071, China; 2120181348@mail.nankai.edu.cn; 2 Centre for Nutrition and Food Sciences, Queensland Alliance for Agriculture and Food Innovation, the University of Queensland, St Lucia, Queensland 4072, Australia; z.shao@uq.edu.au; 3 State Key Laboratory of Food Nutrition and Safety, Key Laboratory of Food Nutrition and Safety, Ministry of Education of China, Tianjin University of Science and Technology, Tianjin 300457, China; smbuder@126.com

**Keywords:** acrylamide, 5-hydroxymethyl-2-furfural, HPLC-DAD, simultaneous detection

## Abstract

Acrylamide and 5-hydroxymethyl-2-furfural (5-HMF) are two of the most abundant compounds generated during thermal processing. A simple method for the simultaneous quantitation of acrylamide and 5-HMF was developed and successfully applied in thermally processed foods. Acrylamide and 5-HMF were co-extracted with methanol and then purified and enriched by an Oasis HLB solid-phase extraction cartridge, simultaneously analyzed by high-performance liquid chromatography and detected with a diode array detector, respectively, at their optimal wavelength. The linear concentration range was found to be 25–5000 μg/L with high linear correlation coefficients (R > 0.999). The limit of detection and the limit of quantitation for acrylamide and 5-HMF were 6.90 μg/L and 4.66 μg/L, and 20.90 μg/L and 14.12 μg/L, respectively. The recovery of acrylamide and 5-HMF in biscuits, bread, Chinese doughnuts, breakfast cereals, and milk-based baby foods was achieved at 87.72–96.70% and 85.68–96.17% with RSD at 0.78–3.35% and 0.55–2.81%, respectively. The established method presents simplicity, accuracy and good repeatability, and can be used for the rapid simultaneous quantitation of acrylamide and 5-HMF in thermally processed foods.

## 1. Introduction

Acrylamide and 5-hydroxymethyl-2-furfural (5-HMF) are two of the most abundant compounds generated during thermal processing and the storage of various foods, especially those rich in protein and reducing sugars [1,2,3]. Some pathways of their formation have been proven to share the first step of the Maillard reaction, which makes acrylamide and 5-HMF a widespread occurrence in daily consumed foods [4,5,6]. On the other hand, 5-HMF, which comes from reducing sugars (especially fructose), can form in many different pathways besides the Maillard reaction, and in a relatively high concentration when compared to acrylamide [7,8].

Acrylamide was classified as probably carcinogenic to humans (Group 2A) by the International Agency for Research on Cancer (IARC) in 1994 (Volume 60) [9]. Furfural was previously reviewed by IARC in 1995 (Volume 63), and 5-HMF gave negative results in standard assays for genotoxicity. 5-sulfoxymethyl-2-furfural, the sulfotransferase-catalyzed metabolite of 5-HMF, is mutagenic [10]. Thus, an Advisory Group of IARC recommended evaluating 5-HMF together with acrylamide and furan [2]. Although, in recent years, 5-HMF was proven to have some positive effects, such as a therapeutic approach for the prevention of IgE-mediated allergic diseases [6] and the significant antioxidant and neuroprotective effects in mice [11], but still, 5-HMF serves as an indicator of quality and security in a wide range of thermally processed foods together with acrylamide [4,8]. Due to their adverse effects and widespread presence in foods, the determination of acrylamide and 5-HMF is required in foods and most of their food processing.

To date, several analytical methods dealing with the analysis of acrylamide have been reported, such as gas chromatography-mass spectrometry (GC-MS) and liquid chromatography-mass spectrometry (LC-MS) [12,13,14,15,16,17], biosensors [18], and microchip electrophoresis [19]. For 5-HMF determination, the dominantly accepted methods were also LC-MS [20,21] and GC-MS [8]. Meanwhile, micellar electrokinetic capillary chromatography (MEKC) [7], liquid chromatography-tandem mass spectrometry equipped with a diode array detector (LC-DAD-MS/MS), nuclear magnetic resonance (NMR) spectrometry [22], capillary zone electrophoresis-tandem ion trap mass spectrometry (CE-MS^2^) [23], and polyoxometalate-coated piezoelectric quartz crystal [21] were also developed for the detection of 5-HMF in diversified food samples. The simultaneous detection of acrylamide and 5-HMF was reported recently, including the LC-MS/MS method [24,25] isotope-dilution HPLC-MS/MS [26], with the limit of quantification being as low as 2.12 ng/mL for acrylamide and 4.86 ng/mL for HMF, which greatly improved the detection efficiency. The above methods are either accurate and sensitive, but with complicated operation and expensive equipment, or fast but inaccurate. A most proper compromise is to use high-performance liquid chromatography (HPLC) which integrates high-separation resolution, accuracy, is less expensive, and is widespread in analytical laboratories. Several detection methods by HPLC have been established and successfully used for acrylamide [27,28,29], and for 5-HMF [30,31,32,33,34], separately. However, to our knowledge, there is no simultaneous methodology for the determination of acrylamide and 5-HMF by HPLC.

The close polarity and ultraviolet absorption character of acrylamide and 5-HMF predicts a probability for a simultaneous extraction, purification, and separation. This study aims to find out a simple approach to the simultaneous quantification method for acrylamide and 5-HMF via HPLC. Different commercial food samples will be analyzed to validate the reliable accuracy and precision regarding the simultaneous detection for acrylamide and 5-HMF, with a much more feasible practice.

## 2. Results and Discussion

To improve the analytical accuracy and precision, the separation conditions of acrylamide and 5-HMF on the HPLC column, the procedures of extraction, the SPE purification and enrichment were all systematically designed and optimized for a simultaneous determination of acrylamide and 5-HMF.

### 2.1. Optimization of HPLC Analytical Conditions

A diode array detector (DAD) was used in this HPLC method, as it not only provides the quantitative results at the fixed wavelength, but can also give the spectrogram of the targets which is helpful for the identification of the targets.

To improve the sensitivity of the analytical method, acrylamide and 5-HMF should be monitored at their maximum absorption wavelength. According to ultraviolet spectra, the maximum absorption of acrylamide and 5-HMF in pure standard solution was observed at 197 nm and 284 nm, respectively. Considering that the high ultraviolet absorption of methanol or acetonitrile (possibly serving as the mobile phase) at 197 nm will increase the background signal of the mobile phase, thus decreasing the sensitivity of detection, and possible impurities in the food samples will have more interference on the detection of acrylamide at a lower wavelength, the detection wavelength of the DAD was thus set, respectively, at 210 nm and 284 nm for the quantitation of acrylamide and 5-HMF. Meanwhile, the spectrogram given by the DAD plays an auxiliary role in the characterization of the targets.

Considering the similar chemical polarity of the analytes and the character of the analytical column ODS C18, the following mobile phases with different elution force were tested, respectively: 100% water, 5% (*v*/*v*) and 10% (*v*/*v*) aqueous methanol solution, and 3% (*v/v*) and 5% (*v/v*) aqueous acetonitrile solution. When 100% water was employed as the mobile phase, excessive peaks and broadening of the solutes was observed due to the poor elution force of water. When various concentrations of methanol and acetonitrile aqueous solutions served as mobile phases, a similar elution and separation effect on acrylamide and 5-HMF was observed. According to the better resolution and lower background noise, 5% (*v/v*) acetonitrile aqueous solution was chosen as the optimum mobile phase.

The flow rate of the mobile phase was also tested. It was found that the increase in flow rate (0.5 mL/min–1.0 mL/min) slightly sharpened the peak area of acrylamide and significantly shortened the retention time. The optimal flow rate was set at 0.6 mL/min; the total chromatographic run-time was 15 min, and the retention time of acrylamide and 5-HMF in the standard solution was about 6.19 min and 12.90 min, respectively.

Under the above optimal conditions, the quantification method of HPLC-DAD for acrylamide and 5-HMF was evaluated including the linearity, limit of detection (LOD), limit of quantification (LOQ), and repeatability. The mixed working standard solutions at six respective concentrations of acrylamide and 5-HMF from 25 μg/L to 5000 μg/L were analyzed, with detection wavelengths of 210 nm and 284 nm. The calibration curves of acrylamide and 5-HMF were obtained by plotting the peak area against the concentration, with a coefficient of determination (R^2^) greater than 0.999. The LOD and LOQ were determined based on the signal-to-noise ratio (S/N) equal to 3 and 10, respectively. The analytical characteristic results of acrylamide and 5-HMF are shown in Table 1.

### 2.2. Co-Extraction of Acrylamide and 5-HMF

Before the application of the established HPLC-DAD analytical method in the simultaneous detection of acrylamide and 5-HMF in thermally processed foods, co-extraction of acrylamide and 5-HMF from the food samples was the key point. This is because one solution should be demanded to extract two targets together, with a high recovery and a low matrix.

The main differences between the reported analytical methods for acrylamide and 5-HMF were the extraction and clean-up procedures. Some of the extraction solutions proposed were compared according to the recovery ratio and the repeatability of results for acrylamide or 5-HMF in previous papers [1,33,34]. Commonly, water is used for acrylamide extraction due to its high solubility in water (215.5 g/100 mL). For the analysis of 5-HMF, methanol is usually used as the extracting solvent. Based on the fact that acrylamide possesses relatively high solubility in methanol (155.0 g/100 mL) and 5-HMF tends to be mutually soluble with both methanol and water, the co-extraction method was optimized using methanol, water and different proportions of both methanol and water as the extracting solutions for a comparative experiment in this study. The results showed that when either water or methanol served as the extraction agent, the chromatographic peak shape and response of 5-HMF on HPLC were not significantly affected. However, for acrylamide, when using methanol as the extract solution, the chromatogram was more stable and the interference of fewer impurities appeared. Biscuits and other food contain complex matrices, especially huge amounts of starch. As such, the water extraction was viscous and hence it was difficult to obtain a clear supernatant after centrifugation. While methanol served as the extraction agent, almost no starch and a number of other macromolecules could be dissolved; therefore, the extraction was clearer than that of water and of the extracting solutions which contained different proportions of both methanol and water. Additionally, methanol can be easily evaporated under a nitrogen gas flow and hence can help to benefit the clean-up operation to follow.

After acrylamide and 5-HMF were both extracted using methanol, Carrez reagents were used to precipitate the matrix soluble in methanol (possibly comprised of proteins, fats, and redox compounds, etc.). It is important to separate the solid residue from the methanol extract before purification, in order to avoid the transfer of the water-soluble food matrix into the methanol extract and burden the following purification step. After the evaporation of the methanol under a nitrogen gas flow, the residue was re-dissolved in water so that the lipophilic matrix was excluded once more as a residue; meanwhile, acrylamide and 5-HMF were completely transferred into the water and then for a further clean-up.

### 2.3. Co-Purification of Acrylamide and 5-HMF by SPE

Most clean-up procedures employed several types of SPE columns as a purification tool for acrylamide determination. A combination of three kinds of cartridges—the Oasis mixed-mode anion exchange, the Oasis mixed-mode cation exchange and the ENVI-Carb—used to purify acrylamide in French-fries samples [35], demonstrated the complexity of the purification of acrylamide in foods. Regarding the 5-HMF purification treatment, different types of commercially available cartridges were compared to improve the precision and purification efficacy, and the ENV+ cartridge was used for the purification of 5-HMF in jam, honey, orange juice, and bakery products matrices before the GC-MS analysis [36]. The Oasis HLB SPE cartridge was used for the clean-up of 5-HMF in baby foods before the quantitation by LC-MS [1]. For the simultaneous detection of acrylamide and 5-HMF, the purification step was alternatively replaced by the isotope dilution method to anticipate the matrix effect from the samples before HPLC-MS/MS, but this was not applicable in HPLC.

In this study, non-polar stationary phase cartridges (HyperSep C18, 3 mL, 200 mg) and hydrophilic–lipophilic balanced copolymer cartridges (Oasis HLB, 6 mL, 200 mg) were evaluated on purification efficacy for both acrylamide and 5-HMF by using their standard solutions to optimize the purification conditions. After the activation of the SPE column, different concentrations of acrylamide and 5-HMF standard solutions were respectively loaded on and flowed through the tested SPE cartridge. 5-HMF was not detected in the effluents from the two SPE cartridges. Relatively, acrylamide had better retention using the Oasis HLB cartridge as it was not detected in the effluent after loading, but 5.2% of the loaded acrylamide was excluded after it was loaded and flowed through the C18 cartridge. It appears that the Oasis HLB cartridge had better retention for both acrylamide and 5-HMF.

To screen the eluting solution for impurities, 1 mL of water was used as the washing solution, considering that both acrylamide and 5-HMF possess a high polarity. The results showed that 5-HMF had good retention on both the Oasis HLB and the C18 cartridge and was not rinsed off the cartridges by the water. However, 19.10% and 84.00% of acrylamide were, respectively, washed off from the Oasis HLB and the C18 cartridge. It is clear that acrylamide tends to adsorb more on the Oasis HLB cartridge than on the C18. This is supported by the fact that the chromatographic packing materials of the Oasis HLB cartridge are of a hydrophilic–lipophilic balance and water-wettable reversed-phase sorbent, while the packing material of the C18 cartridge is based on a high-purity silica gel bonded with inverse phase octadecyl trichlorosilane; hence, the polarity of the carrier in the Oasis HLB cartridge is supposed to be stronger than that of the C18. In addition, acrylamide has a stronger polarity than that of 5-HMF—all the above accounts for the differences in adsorption between acrylamide and 5-HMF on the two kinds of SPE columns.

After the impurity-washing step, the eluting condition was optimized. Methanol was selected as the eluting solvent to elute 5-HMF and acrylamide from the SPE column since it was the good solvent for both acrylamide and 5-HMF. The elution volume (1–6 mL) was then tested to obtain the optimal efficiency. More analytes were eluted with the increase of methanol volume up to 5 mL, which enhanced the recovery of targets; however, more than 5 mL of methanol did not elute more analytes and inversely increased the difficulty of the subsequent enriched processing. Therefore, 5 mL methanol was used as the eluent.

**Figure 1:**
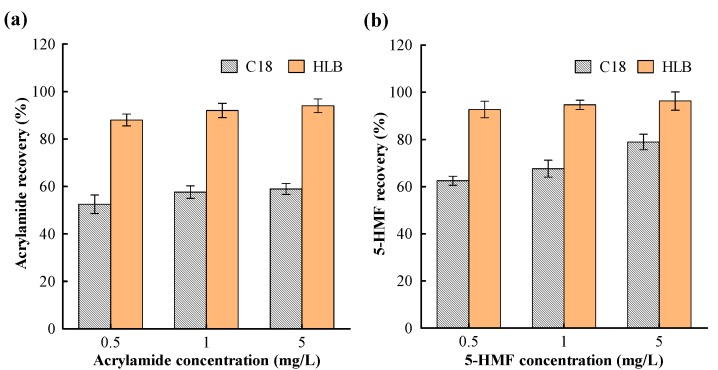
The recovery of acrylamide (**a**) and 5-hydroxymethyl-2-furfural (5-HMF) (**b**) purified by the C18 cartridges and the Oasis HLB cartridges.

Under the optimal purification conditions, the recovery of acrylamide (0.5, 1.0, 5 mg/L) and 5-HMF (0.5, 1.0, 5 mg/L) on the two SPE cartridges was tested (Figure 1). The recovery of acrylamide on the C18 and the Oasis HLB SPE was 52.55–58.90% and 88.70–94.32%, respectively. The recovery of 5-HMF on the C18 and the Oasis HLB SPE was 62.49–78.96% and 92.77–96.23%, respectively. An efficient purification result was achieved on the Oasis HLB SPE which indicates that the Oasis HLB SPE is more suitable for purifying both acrylamide and 5-HMF when compared with the C18 cartridges.

### 2.4. Application and Evaluation of the Established Method in Thermally Processed Foods

**Figure 2:**
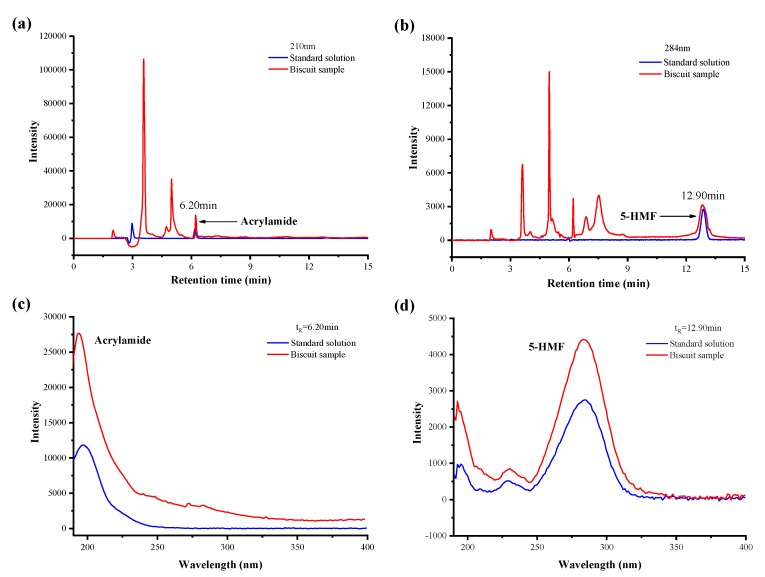
Chromatogram and spectrogram of acrylamide (**a**,**c**) and 5-HMF (**b**,**d**) in standard solution and biscuit samples.

To verify the applicability of the established method in the simultaneous determination of acrylamide and 5-HMF in thermally processed foods, biscuits, bread, Chinese doughnuts, breakfast cereals, and milk-based baby food samples were collected and applied to evaluate the established method. Acrylamide and 5-HMF in the samples were both successfully determined using the developed method under the optimum conditions, including the co-extraction, co-purification, and the HPLC-DAD analytical conditions. Figure 2 shows the chromatogram and spectrogram of acrylamide and 5-HMF in the standard solution and the biscuit samples via the separation on HPLC. The spectrogram of acrylamide and 5-HMF from the biscuit was the same as that of the standard solution. This is indicative of the satisfactory purification effect as no matrix effect remained after the sample purification; meanwhile, the spectrogram of the targets at their retention time benefited from the characterization of the targets.

The determination efficiency was determined by adding acrylamide and 5-HMF standards with three different concentrations in the tested samples (Table 2). The recovery of acrylamide ranged between 87.72% and 96.70%, and the recovery of 5-HMF ranged between 85.68% and 96.17%. The intra-laboratory reproducibility of the method, expressed as the relative standard deviation (RSD, n = 3) was determined with those five food samples and equated to 0.78–3.35% for acrylamide and 0.55–2.81% for 5-HMF. These results show that the established method possesses the acceptable accuracy and precision to meet the demand for the performance of the chromatographic quantitative analysis method.

## 3. Materials and Methods

### 3.1. Reagents and Materials

Acrylamide and 5-HMF were both obtained from Dr. Ehrenstorfer (Augsburg, Bavaria, Germany). Methanol and acetonitrile were of HPLC grade and obtained from Merck (Darmstadt, Germany). Potassium hexacyanoferrate and zinc sulfate were analytical grade and purchased from Sigma-Aldrich (St. Louis, MO, USA). Ultra-pure water used throughout the experiments was prepared by the Milli-Q system (Millipore, Bedford, MA, USA). The Oasis HLB (6 mL, 200 mg) and the HyperSep C18 (3 mL, 200 mg) SPE cartridges were purchased from Waters (Milford, MA, USA) and Thermo (San Jose, CA, USA), respectively. The analytical ODS Hypersil column (250 mm × 4.6 mm, 5 μm) was obtained from Thermo (San Jose, CA, USA). The food samples (biscuits, bread, Chinese doughnuts, breakfast cereals, and milk-based baby food) were obtained from a local supermarket to verify the accuracy of the analytical method.

### 3.2. Standards and Solutions Preparation

Mixed stock solutions of acrylamide and 5-HMF (respective at 1.00 g/L) were prepared by dissolving the precisely weighed standards in ultra-pure water. Working standards solutions (25, 50, 100, 500, 1000 and 5000 μg/L) were prepared by diluting the stock solution with ultra-pure water. Stock solutions and working solutions were, respectively, kept at −20 °C and 4 °C until analysis, and kept for no more than a month and one day in storage.

Carrez I solution was 15% (*m/v*) of aqueous potassium hexacyanoferrate solution and Carrez II solution was 30% (*m/v*) of aqueous zinc sulfate solution—both were prepared according to the previous report [29].

### 3.3. Instruments

HPLC-DAD analyses were performed by an LC-20AT series HPLC system equipped with an SPD-M20A DAD (Shimadzu, Kyoto, Japan). A refrigerated centrifuge 5804R (Eppendorf, Hamburg, Germany) was used for the sample preparation. An SPE system Visiprep DL (Supelco, Bellefonte, PA, USA) and an MNT-2800D pressure blowing concentrator (Auto Science, Tianjin, China) were applied in the sample purification and enrichment procedure.

### 3.4. Extraction of Acrylamide and 5-hydroxymethyl Furfural

Preparation of the samples was carried out according to the previous method with some modification [37]. Two grams of finely triturated samples was weighed into 15 mL centrifuge tubes, added with 5mL of methanol then vortex vigorously for 2 min. The extractives were centrifuged at 11,000× *g* and 4 °C for 10 min. Then, clear supernatant was transferred into a centrifuge tube and added subsequently with 0.25 mL of Carrez I solution and 0.25 mL Carrez II solution. After centrifugation at 11000× *g* and 4 °C for 10 min, 1.0 mL of clear supernatant was transferred into a conical-bottom glass tube and dried with a nitrogen pressure blowing concentrator at 40 °C. The remaining residue was immediately re-dissolved with 1 mL of water for further SPE purification and enrichment.

### 3.5. Purification and Enrichment of Acrylamide and 5-HMF

Before purification, the Oasis HLB SPE column was preconditioned with 5 mL of methanol and 5 mL of water sequentially. Then, 1 mL of the aqueous sample extract was loaded on the cartridge at a rate of one drop per second. Finally, the target analytes were eluted by 5 mL methanol with the same rate of loading as the sample. The first 10 drops of the effluent were discarded to prevent any dilution of the effluent and the following effluent was collected and dried under nitrogen. Then, the residue was re-dissolved in 1mL methanol and filtered through a 0.45 μm filter for quantitation by HPLC-DAD.

### 3.6. Method Recovery in Food Sample

For the recovery study, the food sample was spiked, respectively, with acrylamide (100 μg/kg, 200 μg/kg and 500 μg/kg) and 5-HMF (100 μg/kg, 500 μg/kg and 1000 μg/kg), followed by the extraction and purification procedure as above.

### 3.7. HPLC-DAD Analysis

The C18 ODS Hypersil column (250 mm × 4.6 mm, 5 μm) was used for the chromatographic separation of acrylamide and 5-HMF; the mobile phase was 5% (*v/v*) aqueous acetonitrile solution at 0.6 mL/min and the column oven temperature was 40 °C. The injection volume was 10 μL and the detector wavelength was set at 210 nm and 284 nm. The peak identification was based on the retention time and the ultraviolet spectrum was compared with the standard compounds.

### 3.8. Statistical Analysis

All of the data reported in means ± the standard deviation were statistically calculated from three independent experiments. Analyses were performed using IBM SPSS for Windows (Version 19.0, 2010, SPSS Inc., Chicago, IL, USA).

## 4. Conclusions

Taking advantage of the similar polarity of acrylamide and 5-HMF, an HPLC-DAD analytical method for the simultaneous detection of acrylamide and 5-HMF based on the co-extraction and co-purification has been established. The high efficiency, accuracy, and precision of the developed methodology has been confirmed in the diversified commercial foods. DAD was used in this HPLC method, which not only provides the quantitative results at the dual wavelengths but also the ultraviolet spectrum can provide some auxiliary discrimination on targets. Although the developed method was not as sensitive as the reported HPLC-MS/MS or GC-MS methods, it is still suitable for the detection of acrylamide and 5-HMF since the two compounds were usually generated at high levels in thermally processed foods. In conclusion, the advantages of simplicity, ease of operation, less expense, high accuracy, and precision make the established HPLC-DAD analytical method applicable for the rapid simultaneous quantitation of acrylamide and 5-HMF in a variety of thermally processed foods.

## Figures and Tables

**Table 1:** Linear equations, coefficient of determination, limit of detection (LOD), and limit of quantification (LOQ).

Standard	Linear Equation	R^2^	Linear Range (μg/L)	LOD (μg/L)	LOQ (μg/L)
Acrylamide	Y = 100661.2X + 2159.18	0.9999	25–5000	6.90	20.90
5-HMF	Y = 95429.02X − 817.05	0.9999	25–5000	4.66	14.12

**Table 2:** The recoveries of acrylamide and 5-HMF in different food samples using the HPLC-DAD method (n = 3).

Sample.	Acrylamide					5-HMF				
	Original Concn. (μg/kg)	Spiked Concn. (μg/kg)	Detected Concn. (μg/kg)	Recovery (%)	RSD (%)	Original Concn. (μg/kg)	Spiked Concn. (μg/kg)	Detected Concn.(μg/kg)	Recovery (%)	RSD (%)
**Biscuits**	378.6 ± 4.38	100	467.4 ± 6.75	88.84	1.44	739.75 ± 14.89	100	829.4 ± 8.36	89.67	1.01
		200	562.8 ± 9.61	92.14	1.71		500	1220.6 ± 15.1	96.17	1.24
		500	862.1 ± 6.73	96.70	0.78		1000	1686.9 ± 18.2	94.72	1.08
**Bread**	186.2 ± 5.40	100	275.8 ± 3.84	89.69	1.39	1885.67 ± 16.52	100	1971.4 ± 15.8	85.68	0.80
		200	375.9 ± 5.81	94.87	1.55		500	2351.8 ± 12.9	93.22	0.55
		500	666.3 ± 9.91	96.03	1.49		1000	2813.3 ± 18.6	92.76	0.66
**Chinese Doughnut**	173.3 ± 7.11	100	264.1 ± 8.86	90.78	3.35	419.42 ± 14.25	100	507.6 ± 6.88	88.22	1.36
		200	348.8 ± 7.22	87.72	2.07		500	894.6 ± 7.04	95.03	0.79
		500	633.2 ± 6.88	91.97	1.09		1000	1359.8 ± 9.92	94.04	0.73
**Breakfast Cereals**	284.8 ± 4.11	100	376.1 ± 5.09	91.29	1.35	1166.50 ± 14.51	100	1253.3 ± 10.3	86.75	0.82
		200	473.8 ± 3.73	94.48	0.79		500	1626.8 ± 11.6	92.07	0.71
		500	762.5 ± 6.92	95.54	0.91		1000	2109.8 ± 12.7	94.33	0.60
**Milk-Based Baby Food**	100.2 ± 0.69	100	192.5 ± 5.07	92.31	2.63	194.58 ± 5.28	100	287.9 ± 8.10	93.35	2.81
		200	290.1 ± 3.16	94.92	1.09		500	648.6 ± 8.61	90.80	1.33
		500	579.1 ± 8.17	95.77	1.41		1000	1114.4 ± 13.2	91.98	1.18
